# *Bordetella pertussis* population dynamics and phylogeny in Japan after adoption of acellular pertussis vaccines

**DOI:** 10.1099/mgen.0.000180

**Published:** 2018-05-17

**Authors:** Aldert Zomer, Nao Otsuka, Yukihiro Hiramatsu, Kazunari Kamachi, Naoko Nishimura, Takao Ozaki, Jan Poolman, Jeroen Geurtsen

**Affiliations:** ^1^​ Department of Infectious Diseases and Immunology, Faculty of Veterinary Medicine, Utrecht University, Utrecht, The Netherlands; ^2^​ Department of Bacteriology II, National Institute of Infectious Diseases, Musashimurayama, Tokyo, Japan; ^3^​ Department of Pediatrics, Konan Kosei Hospital, Takaya-cho, Konan, Aichi, Japan; ^4^​ Janssen Vaccines and Prevention B.V., Leiden, The Netherlands; ^†^​ Present address: Department of Molecular Bacteriology, Research Institute for Microbial Diseases, Osaka University, Suita, Osaka, Japan.

**Keywords:** *Bordetella pertussis*, pertactin, phylogenomics, acellular vaccine, epidemiology, Japan

## Abstract

*Bordetella pertussis*, the causative agent of whooping cough, has experienced a resurgence in the past 15 years, despite the existence of both whole-cell and acellular vaccines. Here, we performed whole genome sequencing analysis of 149 clinical strains, provided by the National Institute of Infectious Diseases (NIID), Japan, isolated in 1982–2014, after Japan became the first country to adopt acellular vaccines against *B. pertussis*. Additionally, we sequenced 39 strains provided by the Konan Kosei Hospital in Aichi prefecture, Japan, isolated in 2008–2013. The genome sequences afforded insight into *B. pertussis* genome variability and population dynamics in Japan, and revealed that the *B. pertussis* population in Japan was characterized by two major clades that divided more than 40 years ago. The pertactin gene was disrupted in about 20 % of the 149 NIID isolates, by either a deletion within the signal sequence (ΔSS) or the insertion of IS element IS*481* (*prn *:: IS*481*). Phylogeny suggests that the parent clones for these isolates originated in Japan. Divergence dating traced the first generation of the pertactin-deficient mutants in Japan to around 1990, and indicated that strains containing the alternative pertactin allele *prn2* may have appeared in Japan around 1974. Molecular clock data suggested that observed fluctuations in *B. pertussis* population size may have coincided with changes in vaccine usage in the country. The continuing failure to eradicate the disease warrants an exploration of novel vaccine compositions.

## Data Summary

1. The genome sequence reads of all 188 *B. pertussis* strains were deposited in the European Nucleotide Archive at www.ebi.ac.uk/ under project number PRJEB18624.

2. All supporting data are accessible as supplementary tables. Data include: (1) metadata, sequencing statistics and typing results on 188 Japanese *B. pertussis* strains (Table S1, available in the online version of this article); (2) metadata and typing results on 943 *B. pertussis* strains from this study and the literature (Table S2); (3) sequences and accession numbers of major *B. pertussis* antigen alleles utilized during *in silico* phenotyping of sequenced strains (Table S3); (4) all SNPs detected among the 188 contemporary Japanese *B. pertussis* isolates and strain Tohama I (Table S4); (5) SNPs overrepresented in isolates with *prn *:: IS*481* and/or *prn*ΔSS mutations (Table S5); and (6) rates of genetic variants observed for the orthologous groups of core genes from 188 *B. pertussis* isolates from Japan, 1982–2014, and Tohama I (Table S6).

**Impact Statement**

Japan was the first country to adopt acellular vaccines against *Bordetella pertussis*, the causative agent of whooping cough. After switching from whole-cell to acellular vaccines, a resurgence in the incidence of pertussis in many Western countries, including Japan, was observed. Whole genome sequencing of *B. pertussis* strains harvested across this country identified the *B. pertussis* population dynamics in this environment. In addition to the existence of strains belonging to two different clades that diverged over 40 years ago, clonal spread of pertactin-negative strains was observed in Japan. Our data revealed that these isolates may have arisen in Japan around 1990. We also show a potential direct impact of changes in vaccine usage on the *B. pertussis* population size in the country, and present data on highly variant and very stable bacterial proteins that may aid the development of novel vaccine formulations to better fight the disease.

## Introduction

*Bordetella pertussis* is a non-motile, aerobic, Gram-negative coccobacillus that causes whooping cough (pertussis) in humans. Pertussis is a highly contagious disease of the respiratory tract that, in rare cases, can be fatal, particularly in neonates. Fortunately, vaccines have been developed that reduce disease impact and limit spread of the bacterium in the human population.

More than 100 years ago, whole-cell vaccines (WCVs) against *B. pertussis* were licensed in the USA [1], and were subsequently adopted in many industrialized countries as a combinatorial mixture with diphtheria and tetanus toxoids. In the mid-1970s, increasing reports of adverse reactions to WCV preparations in Japan resulted in a government decision to temporarily halt mass *B. pertussis* vaccination in 1975 and to increase vaccination age after reintroduction of the programme [2]. As a consequence, the development of safer vaccination alternatives was prioritized, and in 1981 Japan was the first country to develop acellular pertussis vaccines (ACVs) and to adopt these for use in the general population [2, 3]. The ACVs invariably contained inactivated pertussis toxin and filamentous haemagglutinin, and were often supplemented with *fim2*-encoded fimbriae and pertactin, the latter an outer membrane protein that promotes adhesion to human tracheal epithelial cells of the host [4]. ACVs helped regain control over pertussis disease in Japan [5], and were shown to be as protective as whole cell-based vaccine preparations [6].

Despite growing vaccine coverage, worldwide incidence of pertussis has increased in recent years. Possible explanations for this resurgence include (i) genetic changes in circulating *B. pertussis* strains (see below), (ii) waning of vaccine-induced immunity, which may be rectifiable by repeated vaccination [7–11], (iii) increased awareness and reporting of pertussis cases, and (iv) an improved diagnosis of pertussis disease (summarized in [12]). In addition, studies in baboons suggest that ACVs, while protecting against disease, have limited impact, if any, on infection with and transmission of *B. pertussis* [13, 14].

Both pre-dating and after ACV development, genomic variations have been observed for ACV-targeted antigens [9, 15–17], some of which may be the result of vaccine-induced selective pressure. A mutation in the promoter region of the operon encoding the pertussis toxin, *ptxP3*, resulted in production of elevated levels of the toxin [18], and now predominates in many countries where ACVs have been deployed [19–22]. Similarly, the non-vaccine pertactin allele *prn2* has become prevalent in many industrialized countries, including Australia, Japan, the UK, the Netherlands, Canada, Austria and the USA [20, 22–27].

Of particular interest is the reported increase in *B. pertussis* isolates not expressing pertactin at all (Prn^−^), observed in many countries that have adopted ACVs [21, 28–35]. The rate of Prn^−^ isolates significantly correlates with vaccine use in the USA [33]. In Japan, the first known pertactin-deficient (Prn^−^) *B. pertussis* strain was harvested in 1997, marking the beginning of an increase of the fraction of pertactin-negative strains in the Japanese *B. pertussis* population to over 30 % during the first decade of the 21st century [36, 37]. The loss of pertactin by some *B. pertussis* strains does not influence disease severity [38, 39]. However, these isolates show increased fitness and/or prolonged infection times in animal host populations immunized with ACVs [40, 41]. Notably, an increased prevalence of Prn^–^ strains is often reported in countries that use pertactin-comprising ACVs for general vaccination [21, 28–30, 32, 35]. However, a recent study revealed a surprising decrease in *B. pertussis* Prn^–^ frequency in Japan in the past 3 years [42].

Many *B. pertussis* genomes have been sequenced and deposited in public repositories. Genomes of the strains harvested during the pertussis epidemic in Australia of 2008–2012 revealed microevolution events, predominant *prn2* alleles and mixing of strains from a concurrent epidemic in the UK [43]. Genome sequencing performed on strains of that UK outbreak revealed that the genes encoding antigens present in ACVs may evolve at a faster rate than other surface proteins of the bacterium, potentially suggesting vaccine-induced selective pressure [25]. Genome sequencing confirmed clonal expansion of *B. pertussis* on four occasions in the Netherlands [26] and suggested differences in the mutation rates of the bacterium depending on vaccination coverage and method in China, the Netherlands and Finland [44]. An extensive study examined and characterized the global population structure of *B. pertussis*, sequencing over 300 isolates, harvested worldwide between 1920 and 2010 [16]. This study included 17 strains collected in Japan between 1988 and 2007, but seemed not to sample any Prn^−^ isolates.

The aim of this study was to further monitor and analyse the *B. pertussis* population dynamics in Japan as the first country to adopt ACV vaccination. To do this, a set of representative *B. pertussis* clinical isolates collected between 1982 and 2014 was analysed by whole-genome sequencing. Our hope was to pinpoint the selection of the first generation of Prn^−^ strains, and to put these into an evolutionary context of currently circulating Prn^–^ and Prn^+^ strains, in Japan.

## Methods

### Selection of *B. pertussis* clinical isolates

A set of Japanese *B. pertussis* isolates was obtained from the Konan Kosei Hospital (*n*=39), representing all *B. pertussis* cases in that hospital between 2008 and 2013. In addition, isolates were procured from the National Institute of Infectious Diseases of Japan (NIID; *n*=151, genome sequences were obtained from 149 of these), representing a random pool of clinical isolates collected from various locations in Japan between 1982 and 2014. A summary of strain characteristics is given in Table 1, while a more detailed description of every strain and associated metadata is given in Table S1. The isolates originated from 29 different locations across all five major islands of the country (Hokkaido, Honshu, Shikoku, Kyushu and Okinawa).

**Table 1.** Summary of Japanese *B. pertussis* strains investigated by whole genome sequencing

Period	Total no. of isolates*	No. of locations	No. of ∆*prn* isolates	∆*prn* mechanisms	Identified gene alleles					
					*prn*	*ptxP*	*ptxA*	*fim2*	*fim3*	*fhaB*
1981–1985	9	2	0	mRNA↓†	1	1	2	1	1	1
1986–1990	13	5	0	–	1	1, 8	1, 2	1	1	1
1991–1995	18	7	0	–	1, 2	1, 3	1, 2	1	1	1
1996–2000	42	11	8	*prn*∆SS, *prn *:: IS*481*	1, 2	1, 3	1, 2	1	1	1
2001–2005	23	16	10	*prn*∆SS, *prn *:: IS*481*	1, 2	1, 3, 8	1, 2	1	1, 2	1
2006–2010	43 (19)	15	23	*prn*∆SS, *prn *:: IS*481*	1, 2	1, 3	1, 2	1	1, 2, 4	1
2011–2014	40 (20)	12	6	*prn*∆SS	1, 2	1, 3	1, 2	1	1, 2, 4	1

*The number of isolates from Konan Kosei is shown in parentheses.

†One isolate exhibited reduced *prn* mRNA production.

### Genome sequencing and assembly

Whole genome sequencing and assembly were performed by BaseClear B.V. Paired-end sequence reads (2×125 bp) were generated using the Illumina HiSeq2500 system. FASTQ sequence files were generated using the Illumina Casava pipeline version 1.8.3. Initial quality assessment was based on data passing the Illumina Chastity filtering. Subsequently, reads containing adapters and/or PhiX control signal were removed using an in-house filtering protocol. The second quality assessment was based on the remaining reads using the FASTQC quality control tool version 0.10.0. The quality of the FASTQ sequences was enhanced by trimming off low-quality bases using the ‘Trim sequences’ option of Qiagen’s CLC Genomics Workbench version 7.5 or 8.0. The quality-filtered sequence reads were assembled into contigs, using SPAdes v3.10.1 with default settings, including the *careful* mode. Final genome coverage and contig numbers are shown in Table S1.

The genome sequence reads of all 188 *B. pertussis* strains were deposited in the European Nucleotide Archive at www.ebi.ac.uk/ under project number PRJEB18624.

### Phylogenetic reconstruction

Illumina sequence reads or complete genomes of all sequenced *B. pertussis* isolates from this study, as well as qualifying *B. pertussis* genome sequences available from GenBank and those deposited from Sealey and others [25] in the European Nucleotide Archive ENA (755 GenBank and ENA genomes, access date 9 Febuary 2018) were mapped against the closed genome of *B. pertussis* Tohama I [45], using Snippy with default settings [46]. Genomes were only included when coverage was sufficient (defined as fewer than 300 kb with a coverage of <10) and when isolation location and date were provided. Typing data were extracted from various reference sources [16, 25, 45, 47–52] or inferred from sequenced genomes (Table S2). Phylogenetic trees with and without inclusion of the 755 worldwide isolates were reconstructed using FastTree2 [53] with a generalized time-reversible (GTR) model with gamma correction on the resulting core genome alignment. The resulting trees were rooted using the genome of *Bordetella bronchiseptica* MO149 [54] prior to visualization using iTOL [55].

### *In silico* typing and phenotypic analysis of isolates

For *in silico* typing, alleles of the genes *ptxA*, *ptxP*, *fim2*, *fim3*, *prn* and *fhaB* were aligned against assembled whole genome sequences of the isolates using blast +2.4.0 (ftp://ftp.ncbi.nlm.nih.gov/blast/executables/LATEST). A gene was assigned an allele if a 100 % match was found against the polymorphic typing region, as previously defined [56, 57]. Apparent mutations were manually verified from the sequence alignments. The numbers of alleles considered were as follows: *prn*, 17 alleles, *ptxP*, 11 alleles, *ptxA*, 3 alleles; *fim2*, 2 alleles; *fim3*, 6 alleles; *fhaB*, 2 alleles. Alelle sequences and accession numbers are shown in Table S3. We used assembly data for this analysis, as well as for SNP and gene variant detection (below), because the use of mapped reads may disable detection of insertions and deletions.

Phenotypic analyses for expression of Prn, Fim2 and Fim3 were performed as previously described [36], using immunoblotting of 1 µg protein samples per lane (Prn) and whole-cell ELISA techniques (Fim2, Fim3).

### Divergence dating and recombination analysis

A core genome alignment was created on the isolates from this study using Parsnp v1.2 [58], and SNPs were detected from the alignment. SNPs closer than 1 kb to each other were excluded to remove rapidly evolving regions or regions that are under selective pressure which could affect the temporal signal. This approach resulted in removal of 400 SNPs from consideration. Pearson correlation between root to tip distance of the phylogenetic tree and isolate date was significant (*P*<0.0001, *R*^2^=0.38), suggesting a strong temporal signal in the SNP data. Divergence dating was performed in beast [59], using the isolation dates as tip dates in the phylogenetic tree with a GTR model of evolution. An exponential clock model was used in combination with a Bayesian skyline demographic model with four groups, essentially as previously described [16]. A Markov chain was run for 100 000 000 generations, with parameter values sampled every 10 000 generations. The chain was checked for expected sample sizes (ESSs)>200 using Tracer (http://tree.bio.ed.ac.uk/software/tracer/), with the first 10 000 000 chains removed [59]. A Bayesian skyline plot was drawn in Tracer with dates between 1950 and 2014. A maximum clade credibility tree was computed with TreeAnnotator, keeping tree heights intact and with a burn-in of 10 000 000 chains. beast results were visualized using Tracer and Figtree (http://tree.bio.ed.ac.uk/software/figtree/).

### Detection of SNPs, and association of SNPs with Prn^+^ and Prn^–^ *B. pertussis* isolates

Complete genome sequences of *B. pertussis* isolates were compared using Parsnp v1.2 [58]. Presence and absence of core genome SNPs, as identified by Parsnp, were tested for their prevalence in Prn^−^ and Prn^+^ strains using Fisher's exact 2×2 contingency tests.

### Rates of genetic variants

Proteins were clustered into orthologous groups (OGs) using Roary [60] with default settings. Prior to clustering, genes containing transposase sequences were removed using blast against genes coding for transposases [61], to prevent spurious clustering of proteins on these sequences. Amino acid and nucleotide sequences of these OGs were compared and every unique gene or protein sequence was assigned an allele number. Unique numbers of variants per OG were counted and summarized. Truncated genes shorter than 90 % of their supposed length were removed from the analysis. The computed variant numbers per OG (minus 1) were finally divided by the length of the represented gene to generate a density value that allows cross-comparisons between genes.

Note that a genetic variant is based on the entire ORF sequence, while *prn*, *ptxP*, *ptxA*, *fim2*, *fim3* and *fhaB* alleles only pertain to the typing regions defined for each gene. Consequently, there can be more genetic variants than alleles for these genes.

## Results and discussion

### Phylogenetic analysis of pertussis disease in Japan reveals two major clades

To gain insight into the *B. pertussis* phylogeny and population dynamics in the country that first adopted ACVs, we sequenced the genomes of 149 clinical *B. pertussis* isolates observed across Japan between 1982 and 2014. These strains were provided by the NIID. In addition, we sequenced the genomes of 39 isolates from the Konan Kosei Hospital, a local healthcare facility in Aichi prefecture in Japan, with isolation dates from 2008 to 2013. Overall, about 1.44 million reads (sd±0.3) were obtained per isolate, resulting in an average genome coverage of 88.2× (sd±18.3). Core genome alignment, which included 3 552 899 bp DNA, revealed a total of 1272 SNPs between the 188 isolates and *B. pertussis* strain Tohama I, the strain used for preparation of the ACVs that are utilized in Japan.

**Fig. 1.**
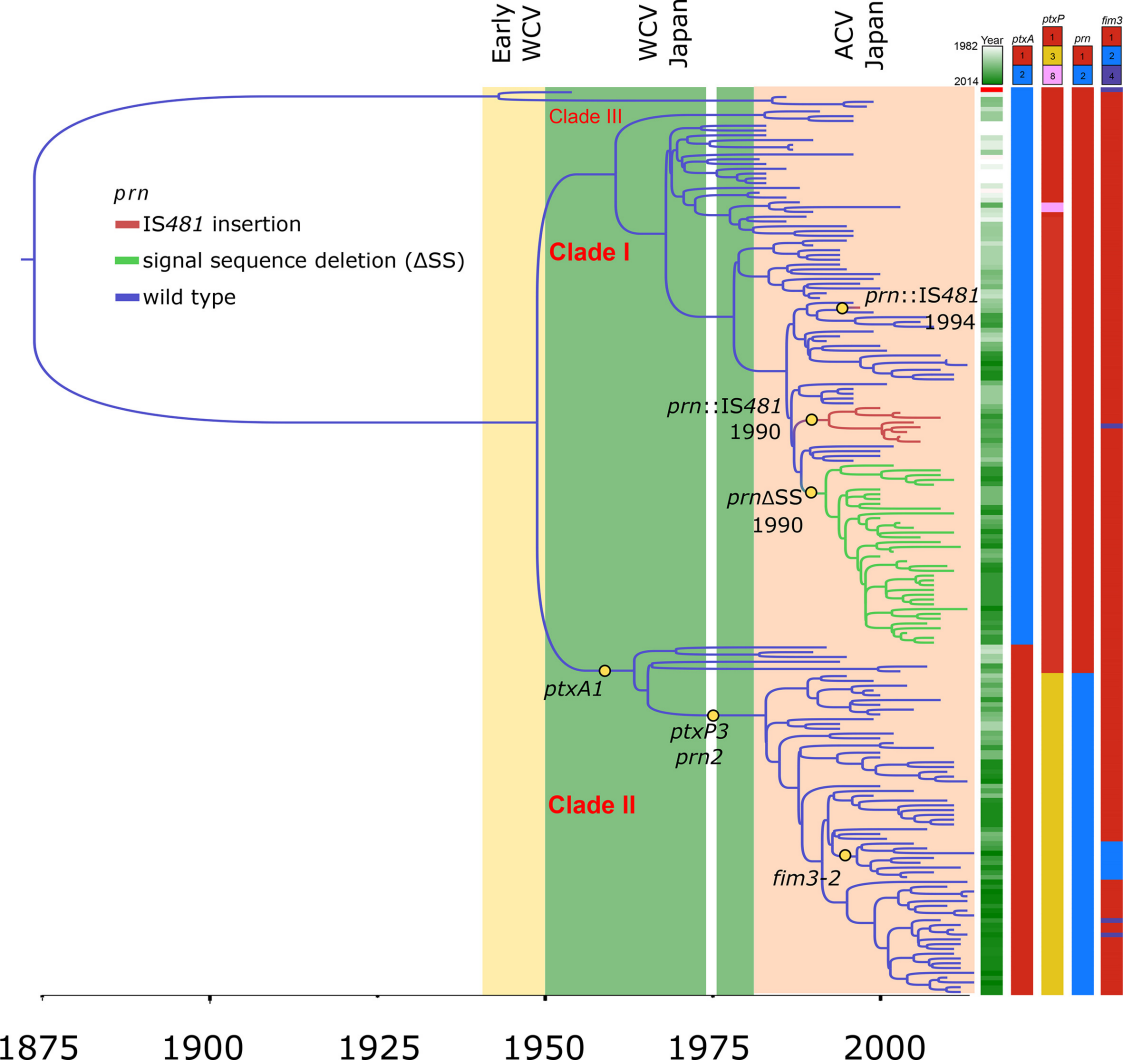
Phylogeny of *B. pertussis* isolates in Japan. Shaded regions indicate time periods of WCV and ACV use, with the notable gap in vaccine use in 1975. Isolation year, alleles of *ptxP*, *ptxA* and *prn*, as well as pertactin deficiency is indicated for each isolate on the right. The clustered isolates of two variants of pertactin-deficient strains are highlighted in red (*prn *:: IS*481*) and green (ΔSS). Notable nodes are illustrated and beast-based divergence dating results are shown for the pertactin-deficient variants.

**Fig. 2.**
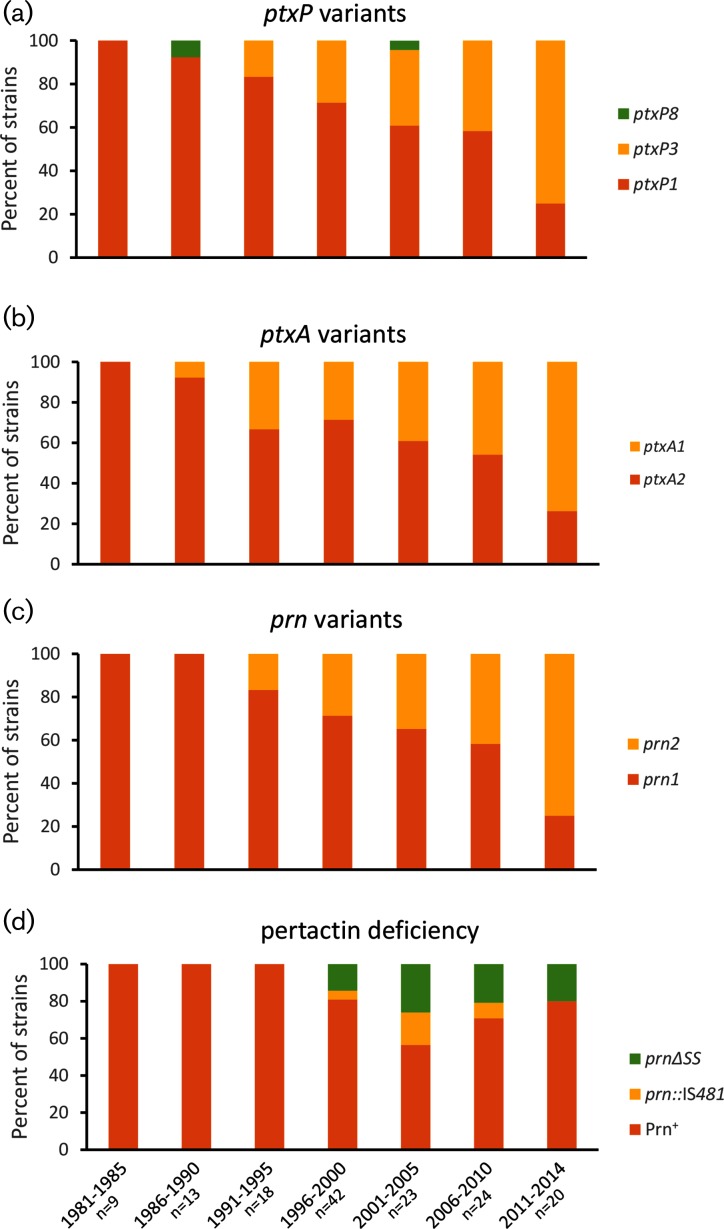
Changes in *B. pertussis* allele frequencies of *ptxP*, *ptxA* and *prn* as well as pertactin deficiency rates over time in Japan, 1982–2014. Konan Kosei isolates were excluded from the calculation.

Phylogenetic reconstruction based on 872 SNPs identified in the core genome present in all sequenced strains revealed that two clades of *B. pertussis* represent the primary causes of pertussis disease in Japan from 1982 to 2014 (Fig. 1). This analysis excluded SNPs that were within 1 kb of each other. The clades are characterized by defined differences in the alleles of major antigens of the bacterium. The first clade consists of 112 isolates predominated by exhibition of pertussis toxin allele *ptxA2*, pertussis toxin promoter allele *ptxP1* and pertactin allele *prn1*. The vast majority of the 73 strains forming the second clade contain the *ptxA1 : ptxP3 : prn2* combination. Median isolation dates reveal the first clade (median isolation year 2000) to contain overall older strains than the second clade (median isolation year 2009), indicating an overall increase in prevalence of strains containing the *prn2* and *ptxP3* alleles. A closer look at the temporal prevalence of different alleles of the *prn* and *ptxA* genes and *ptxP* confirms this observation (Fig. 2a–c).

**Fig. 3.**
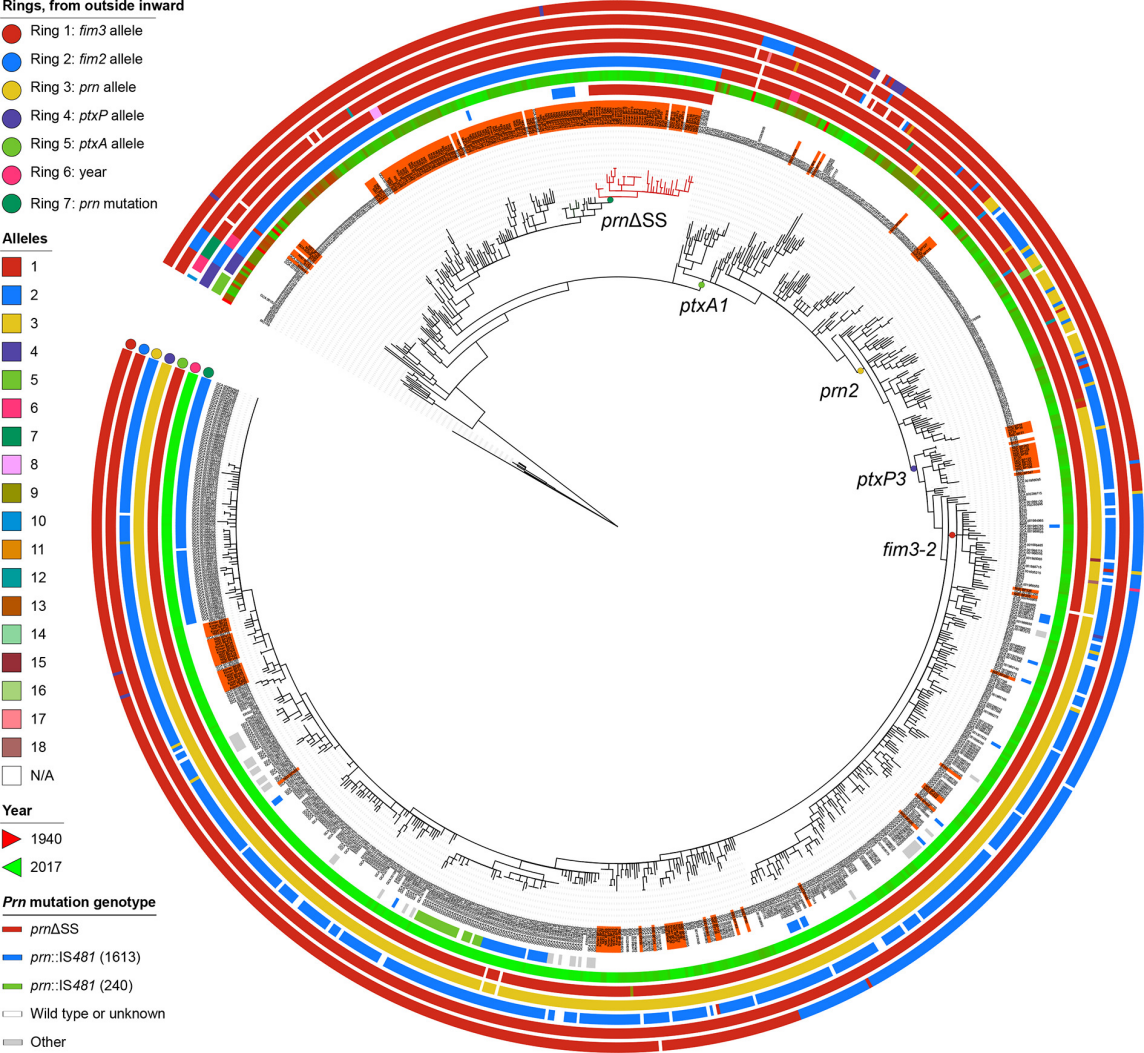
Phylogenetic tree of *B. pertussis* isolates in Japan, contextualized with worldwide isolates. Japanese isolates are on an orange background. Worldwide isolates are those investigated in previous studies [16, 25] and all other qualifying genome sequences deposited in GenBank by 9 February 2018 (see Methods). Alleles of *fim3*, *fim2*, *prn*, *ptxP* and *ptxA* and isolation year are colour-coded, as indicated on the left. The variants of pertactin deficiency are highlighted in blue and green (*prn *:: IS*481*), red (ΔSS) and grey (other). Notable nodes are annotated. Circles represent, from outermost inwards, *fim3*, *fim2*, *prn*, *ptxP* and *ptxA* allele status, followed by isolation year of each strain and *prn* mutation status. Distances in the tree were adjusted to sites per core genome and square root transformed to enhance resolution at short phylogenetic distances.

A minor third clade was also identified, which consisted of reference isolate *B. pertussis* Tohama I (*ptxA1 : ptxP1 : prn1*) and three additional isolates (BP56, BP121 and BP194), all harvested before 2000. The population structure in Japan broadly mirrors observations made in other countries. However, we noted one minor distinction. A separate clonally expanding clade that included *ptxA1 : ptxP3 : prn2* strains with the *fim3-2* allele was detected in the UK and the Netherlands in the early 2000s [25, 26]. A small *fim3-2* clade of eight isolates with the same antigenic combination was also present in Japan, but has not expanded to the same predominance and clusters deep within clade II (Figs 1 and 3).

### Comparison with previously sequenced isolates and worldwide context

Extensive earlier work sequenced *B. pertussis* genomes obtained worldwide, to gain insight into evolutionary patterns on a global scale [16]. This study included 17 strains harvested from Japan between 1988 and 2007 and pertactin deficiency had not been recognized in the genome assemblies for any of these strains. However, an earlier report had suggested lack of pertactin in one of these isolates, strain BP310 [36]. We therefore re-sequenced nine of these 17 strains (BP22, BP120, BP128, BP121, BP31, BP56, BP119, BP227, BP310), and confirmed a ΔSS pertactin phenotype in strain BP310. After assembly of the reads from all 346 isolates included in the earlier study [16], previously unnoticed ΔSS pertactin genotypes were also noted in two further strains, B050, harvested in 2007 in Australia, and B107, a 2002 isolate from Hong Kong. A phylogenetic placement of our panel of Japanese isolates with the strains sequenced as part of the worldwide investigation in 2014 provided a worldwide context for the Japanese isolates. This context was expanded to include all other qualifying *B. pertussis* genome sequences in GenBank and ENA (see Methods, Fig. 3), for a total of 943 genomes. Among other observations, the tree indicated that three additional isolates, harvested in 2010–2012 in the USA, clustered with the Japanese *prn*ΔSS isolates and were possibly introduced into North America from Japan.

The Japanese isolates populated all major branches of the global tree, indicating that the parameters used for tree reconstruction minimized any systematic difference introduced by the distinct sequencing approaches for the different strains. However, no Japanese isolate was identified that populated the intermediate evolutionary clade after the appearance of the *prn2* allele but pre-dating the introduction of the *ptxP3* mutation, i.e. *ptxP1 : prn2*.

We observed strong geographical clustering of the Japanese *ptxA2* isolates, suggesting that they had evolved there for a prolonged amount of time and remained clonal. This was unexpected, given the lack of geographical clustering of worldwide *B. pertussis* isolates observed elsewhere [16]. However, the tree intimated a few Japanese isolates to have been introduced into the country from elsewhere, by placing these in close proximity to older strains from the worldwide panel. These ‘island’ clones are exemplified by BP28, a strain that closely resembles B250, a Swedish isolate harvested in 1970, and isolates BP227 and BP316, which are similar to B014, a strain harvested in 1971 in Australia, and two European strains isolated in 1968 (B071 from Denmark and B221 from Poland). Conversely, a few strains found in the global strain panel had very close relatives in the Japanese strain panel, such as the previously mentioned three USA strains clustering with the *prn*ΔSS isolates.

Three isolates from our study, BP56, BP194 and BP121, two Japanese isolates from the worldwide panel (B133 and B135, harvested in the late 1990s), and one additional Japanese isolate from 1950 (GCA_002083095) clustered in an outgroup with the model isolate Tohama I, and represent a remote branch that has become extremely rare and possibly extinct in modern Japan. On a worldwide scale, this small cluster included a few other older strains, such as J042, an isolate from 1947 isolated in the USA, as well as B068 and GCA_001605275, slightly more distant Chinese isolates from the 1950s.

### Allele typing

*In silico* typing was performed on all sequenced isolates for major antigens of *B. pertussis*, i.e. *ptxA*, *fim2*, *fim3*, *prn* and *fhaB*, as well as the pertussis toxin promoter region *ptxP*. Results are shown in Table 1 and, in single-strain resolution, in Table S1. For *ptxA*, two types were found, *ptxA1* (*n*=73) and *ptxA2* (*n*=115), separated along clade lines. For *ptxP*, three types were found, *ptxP1* (*n*=119), *ptxP3* (*n*=67) and *ptxP8* (*n*=2). All *ptxP3* isolates and 6/119 *ptxP1* isolates constituted clade II of the phylogenetic tree shown in Fig. 1, while two *ptxP1* strains clustered with the type strain Tohama I in the small and possibly extinct clade III. The remaining 111 *ptxP1* strains and the two *ptxP8* isolates formed clade I. All strains contained *fim2-1* (*n*=188). For *fim3*, three types were found, *fim3-1* (*n*=177), *fim3-2* (*n*=8) and *fim3-4* (*n*=3). For *fhaB*, only *fhaB-1* was detected (*n*=188).

Based on previously defined amino acid repeat variations in pertactin sequences [57], two *prn* types were genotypically identified in the strain panel, *prn1* (*n*=121) and *prn2* (*n*=67). Among the *prn1* strains, 38 had a deletion in their 5′ signal sequence-encoding region (ΔSS) and nine were interrupted by an IS*481* element (*prn *:: IS*481*) in their *prn* gene.

### Allelic shifts and deficiency of pertactin

A decline in *prn1* frequency, paralleling ascending *ptxA1* and *ptxP3* prevalence, was observed in existing isolates in Japan, a shift that experienced its biggest momentum at around 2010 (Fig. 2a, b). These events mirror similar shifts toward alternative antigen alleles observed earlier on a worldwide scale, primarily in the industrialized world with high vaccine coverage. The start of this worldwide shift pre-dated use of ACV preparations and suggested exertion of selective immune pressure on pertactin by WCVs. Consequently, allelic variation of *B. pertussis* antigens probably contributed to survival of *B. pertussis* after WCV adoption, as previously championed [16].

The vaccine strain Tohama I expresses *prn1* and *ptxA2*, and the recent shift favours strains expressing antigen variants not included in the ACV preparations customary in Japan. Evidence of increasing incidence of pertactin variants not represented in vaccine preparations had been presented before (exemplified by [17, 62]), and similar changes had been described, beginning in the WCV era, on a worldwide scale [16]. However, this observation may be restricted to countries with high vaccine coverage, as countries with lower, or delayed, vaccine coverage have not (yet) experienced similar predominance of *prn2* isolates [44, 63], or are apparently devoid of *prn2* strains [64]. Notably, an allele shift to *prn2:ptxA1* resulted in prolonged survival in murine test animals treated with an ACV derived from the *prn1* isolate Tohama I [65].

Divergence dating using beast on a core genome alignment (see Methods) places the divergence node between *prn2* and *prn1* strains in Japan at around 1974, well within the period of WCV usage, but pre-dating the development of ACVs against *B. pertussis*. This suggests that WCVs induce pertactin-selective immune pressure. Allelic variation of pertactin has been observed in many countries prior to introduction of ACVs, during periods of high WCV coverage [66–68].

Pre-dating the decline in *prn1* predominance, pertactin-deficient isolates appeared in the mid-1990s in Japan. In our strain panel, this deficiency occurred exclusively in *prn1* alleles, in two ways. The first consists of an integration of the insertion sequence IS*481* (in the forward orientation) at nucleotide position 1598 of the *prn* gene (equivalent to nucleotide position 1613 in *prn2*). The second consists of a deletion of nucleotides 26–109 in the signal sequence of the *prn* gene. Both disruption of the gene by IS*481* and signal sequence deletion have been previously observed and described in Japanese *B. pertussis* Prn^–^ isolates [37], and IS*481* insertions, although in different locations within the gene, were also observed elsewhere [30, 32, 34]. In our strain selection, divergence dating estimates the divergence of the *prn*ΔSS cluster in the phylogenetic tree depicted in Fig. 1 to have occurred in 1990 [95 % confidence interval (CI): 1989–1993]. In the same year, a *prn *:: IS*481*-containing mutant cluster may have first appeared (95 % CI: 1989–1995). A lineage that included a third single *prn *:: IS*481* isolate, BP118, was predicted to have diverged from an intact ancestor around 1994 (95 % CI: 1991–1997). Such pertactin-deficient isolates reached a maximal frequency in Japan of over 40 % in the early 2000s (Fig. 2d). The phylogenetic tree including the worldwide strain collection (Fig. 3) does not detect a close ancestor to the Japanese *prn*ΔSS isolates in the worldwide collection, and therefore suggests this cluster to have arisen in Japan. Similarly, no close relative of the *prn *:: IS*481* strains isolated in Japan was detected in the panel of worldwide isolates.

However, Prn^–^ isolates failed to take over the *B. pertussis* population in Japan, and a recent study reveals a decrease in *B. pertussis* Prn^–^ frequency in Japan to below 10 % in the past 3 years [42]. One reason for this drop may be the rising predominance of *prn2*-containing isolates, many of which have close relatives elsewhere in the world (illustrated in Fig. 3). However, this drop in pertactin-negative *B. pertussis* strains may also be caused, in part, by the wide introduction of new ACV preparations in Japan in 2012 that do not contain pertactin [42]. It remains unclear why Prn^−^ isolates thrive to various degrees in different territories, exemplified by their rapidly increasing prevalence in Australia [32, 43] and the USA [69, 70], but continued low frequency in neighbouring Canada and the UK [71]. One explanation assumes that differential vaccine preparations and immunization regimens impose different levels of immune pressure on pertactin, dictating a varying need for and availability of possible escape routes.

While more than a dozen genetic disruption mechanisms of pertactin have been identified, including alternative IS elements (such as IS*1002*), SNPs, premature stop codons and various deletions [21, 32, 70, 72], Prn^−^ strains fail to fully outcompete Prn^+^ isolates. As a case in point, only a single Prn^−^ strain has been identified in a recent study that analysed 95 isolates harvested mostly between 2000 and 2012 in the UK, a country that switched from WCV to ACV for primary immunizations in October 2004 [25, 73]. Therefore, pertactin deficiency in itself, while perhaps initially providing an evolutionary benefit, may not carry sufficient advantages to eradicate other *B. pertussis* isolates, particularly those with a non-vaccine-type *prn* allele, e.g. *prn2*, and/or increased pertussis toxin production afforded by the *ptxP3* mutation.

### Gene variants and SNPs present in isolates with disrupting mutations in the pertactin gene

Genome-wide comparison of gene variants in Prn^+^ and Prn^−^ isolates identified several gene differences present in isolates without functional pertactin. The majority of these associations probably represent hitchhiking mutations that occurred during acquisition of pertactin deficiency, and were clonally retained. Among gene variants, the most significant association was detected for an 11 bp deletion in gene *BP0310* (position 315 417–315 427 in the genome of strain Tohama I), encoding a putative acyl-CoA-dehydrogenase, resulting in a truncated protein (data not shown). This variant was absent from the *prn *:: IS*481* isolates and from the vast majority of Prn^+^ strains but was found in all *prn*ΔSS isolates and in only four Prn^+^ isolates (BP1, BP9, BP61 and BP156). The latter four isolates cluster with the *prn*ΔSS isolates on the phylogenetic tree, perhaps indicating that this deletion preceded the deletion of the signal sequence in *prn*.

Table S4 shows all SNPs identified in this study among all 188 isolates, compared to strain Tohama I, and Table S5 illustrates the results of an analysis of SNP differences between Prn^+^ and Prn^−^ strains of the *ptxA2 : ptxP1 : prn1* clade. We identified a non-synonymous SNP present exclusively and consistently in all *prn*ΔSS strains in gene *BP0534*, encoding a putative enoyl-CoA hydratase/isomerase. No SNPs were found that were present in all *prn *:: IS*481* Prn^−^ isolates but not in Prn^+^ *ptxA2 : ptxP1 : prn1* isolates. However, SNPs in *BP0332* and *BP1640*, both encoding proteins of unknown function, were detected in all but one *prn *:: IS*481* Prn^−^ isolate, and nowhere else. The *prn *:: IS*481* Prn^−^ isolate lacking this SNP was BP118, a strain that clustered separately from all other *prn *:: IS*481* isolates on the phylogenetic trees depicted in Figs 1 and 3. The functional effects of the detected SNPs and gene variants on bacterial pathogenicity, growth and fitness are unknown but may be verified with additional future experiments.

### Local *B. pertussis* population dynamics may differ profoundly from national trends

The 38 strains that contained a deletion of the signal sequence of the pertactin gene were deficient in pertactin production and clustered together on the phylogenetic tree, indicating a clonal spread of the isolate. These strains had been harvested over the course of 13 years, between 2000 and 2013. A total of 18 of these strains had been provided by the Konan Kosei Hospital. Among all *B. pertussis* isolates reported in this hospital, prevalence of this clone reached over 90 % in 2008 and 100 % in 2009, while, according to our strain panel, the same clone represented only around 16 % of *B. pertussis* isolates found in the rest of the country at that time. After 2009, the isolate’s prevalence in Konan fell back to approximately national levels. This observation suggests that this particular *prn*ΔSS isolate prevailed and persisted over at least 2 years within the Konan community. It is therefore likely that the overall fitness of this strain was at least as good as that of standard Prn^+^ isolates that existed in the vaccinated population of Konan at the same time.

### Rates of genetic variants

We performed an examination of the rates of genetic variants (nucleotide variant rates per base pair) observed in the core genome among the sequenced *B. pertussis* isolates from Japan. Table S6 shows all variation rates for each *B. pertussis* Tohama I gene included in the core genome. The analysis confirmed a higher overall rate of variants for the nine genes predicted to encode ACV-targeted proteins (*fhaB*, *fim2*, *fim3*, *prn*, *ptxA–E*), compared to all genes (0.0014 versus 0.0006 variants/bp). A summary of the observed overall rates and the amino-acid-changing rates for the functional categories of the *B. pertussis* Tohama I core genes is depicted in Table 2. The analysis suggests that genes targeted by ACVs do indeed mutate more frequently than coding sequences in most other gene categories, an effect that had been previously described [25]. They also mutate, on average, more frequently than genes encoding proteins predicted to be located extracellularly (20 genes, 0.0006 variants/bp) or in the outer membrane (70 genes, 0.0010 variants/bp). In contrast to previous observations [16], this trend of elevated mutation frequency does not extend to a similar degree from the ACV genes to other virulence-associated genes in our strain panel. Perhaps this can be explained in part by the early adoption of ACVs in Japan, thereby focusing pressures onto the vaccine-targeted proteins earlier than in other countries.

**Table 2.** Rates of genetic variants observed in functional gene categories in Japanese *B. pertussis* isolates

Functional category*	Number of genes	Median gene length	Non-silent variant rate†	Total variant rate‡
Fatty acid metabolism	21	936	0.0002	0.0004
Small molecule degradation	107	1068	0.0003	0.0004
Macromolecule degradation	46	1234.5	0.0003	0.0004
Cell division	18	1014	0.0003	0.0004
Ribonucleotide biosynthesis	27	1080	0.0004	0.0004
Macromolecule synthesis/modification	194	1068	0.0004	0.0005
Cell processes	62	927	0.0004	0.0005
Cofactor biosynthesis	86	846	0.0004	0.0005
Ribosome constituents	55	384	0.0004	0.0005
Amino acid biosynthesis	91	1134	0.0004	0.0005
Adaptation	48	563.5	0.0005	0.0005
Central/intermediary metabolism	96	1016.5	0.0004	0.0005
Energy metabolism	119	1056	0.0004	0.0005
Transport/binding proteins	326	970.5	0.0004	0.0006
Miscellaneous	342	886.5	0.0005	0.0006
Conserved hypothetical	419	681	0.0005	0.0006
Cell surface	571	951	0.0005	0.0006
Protection responses	24	639	0.0006	0.0006
Regulation	270	829.5	0.0007	0.0008
Virulence-associated genes	89	1095	0.0007	0.0009
Unknown	134	463.5	0.0008	0.0010
ACV genes	9	681	0.0012	0.0014
Phage-related or transposon-related	21	648	0.0013	0.0016
**All genes**	3168	879	0.0005	0.0006

*Functional categories are from a previous source [16].

†The variant rate was calculated as (sum of amino acid-changing variants of genes in category – number of genes in category)/(sum of gene length of all genes in category, in bp).

‡The variant rate was calculated as (sum of nucleotide variants of genes in category – number of genes in category)/(sum of gene length of all genes in category, in bp).

A number of genes encoding proteins that localize close to the bacterial surface, or whose localization has not been characterized in detail as yet, display a higher than average genetic variation rate, suggesting that these may have experienced high antigenic pressure. Among the 15 genes that encode seven or more amino acid variants in our strain panel were *fhaB* (11 variants) and two autotransporters, *tcfA* (seven variants) and *sphB2* (31 variants). Further investigation may be warranted to better understand the observed sequence variation in these genes.

The genetic variant analysis also revealed 33 outer membrane proteins, 17 of which are known to be under Bvg control, which displayed only one genetic variant in the 188 Japanese strains (Table S6). These proteins may be of some interest for future strategies to combat pertussis, although strong sequence conservation may be indicative of low immune pressure. Among these are components of several TonB-dependent iron uptake systems, including the putative haem receptor HemC and putative ferric siderophore receptors encoded by the *bfr* operon [74]. The protein that suppresses the bactericidal activity of the complement, BrkA [75], and the putative fimbrial usher protein FimC [76] were also among the gene products with no observed variations. Seven of these genes, including *bfrH* and the gene encoding the outer membrane protein OmpA, were also conserved in 343 worldwide strains [16].

A normalized comparison of previously reported SNP densities from isolates stemming predominantly from the WCV era [16] with the ACV-era variant rates observed in the Japanese strain panel did not reveal significant differences in the propensity for genetic variation in the nine genes encoding ACV-targeted proteins (data not shown).

### Temporal fluctuations of the *B. pertussis* population size in Japan

**Fig. 4.**
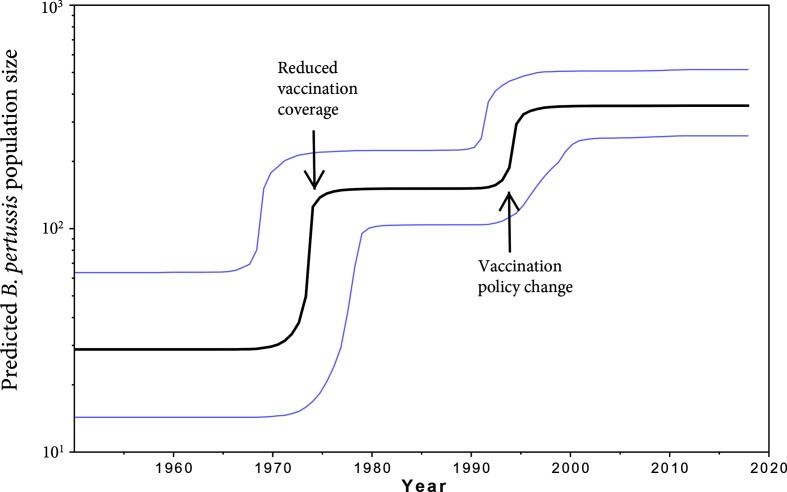
Bayesian Skyline Plot of *B. pertussis* in Japan, excluding Konan Kosei isolates. Fluctuations in the *B. pertussis* effective population size per year relative to the sample data were inferred from the fluctuations in the number of mutations in the *B. pertussis* genomes sequenced. The 95 % CIs are in blue. The predictions are based on the molecular clock data analysis as determined by beast (see Methods). Coinciding events that may affect population size are marked by arrows.

Based on the molecular clock data analysis as determined by beast, fluctuations in the *B. pertussis* effective population size per year relative to the sample data were inferred from the fluctuations in the number of mutations in the *B. pertussis* genomes sequenced. For this analysis, the isolates obtained from Konan Kosei Hospital were excluded. As illustrated in Fig. 4, two increases in effective population size were predicted between 1950 and 2012 – one around 1974, and another increase in the mid-1990s. The first increase coincides with a public debate on vaccine safety following two infant deaths within 24 h after WCV administration in the winter of 1974/75, which led to a 2-month temporary suspension of the use of the vaccine and a subsequent raise in age from 3 months to 2 years at which the primary vaccine dose was to be administrated [77]. These events may explain the inferred increase in *B. pertussis* population size. The second increase coincides with a change in the Preventive Vaccination Law in 1994 in Japan where mass vaccination in regional Public Health Centers was replaced by individual private inoculation upon recommendation, while at the same time the recommended age for the primary series of ACV inoculation was changed from 2 years back to 3–12 months [78, 79]. Consistent with our prediction of an increased *B. pertussis* population size in Japan after temporary cessation and/or a lower vaccination rate, previous studies have detected the reverse effect on population diversity after introduction of vaccine programmes [73, 80, 81]. However, because genome sequencing has higher resolution than the typing methods employed in those studies, the magnitudes of these effects are difficult to compare.

Notably, despite the beast-predicted increase and subsequent stabilization of the effective population size of *B. pertussis* in the mid-1990s in Japan, the actual case numbers reported to the NIID declined in the late 1990s [82, 83]. ACVs may therefore effectively protect against developing pertussis disease, but may not prevent asymptomatic carriage and circulation of the bacterial strains, as observed in vaccination studies in baboons [13].

In Japan, as in other parts of the world, pertussis has experienced a worldwide resurgence that cannot be attributed to lower vaccine coverage alone. The rising percentage of pertussis cases in Japanese teenagers or adults [84] suggests waning immunity to be one contributing factor, coupled with a vaccination schedule that incorporates a booster at an early age (2 years) but lacks a secondary immunization for adolescents, as championed in the USA [2]. Recent studies revealed that the level of decrease in vaccine effectiveness against pertussis disease depends on both the specific vaccine antigen and the subject’s age [85]. As a short-term solution, the efficacy of existing vaccines may be improved by a change in vaccination booster regimens, by an expansion of maternal immunizations or by improving existing ACV antigens. However, novel targets for future ACV approaches, including a potential change in adjuvants, may be desirable [86, 87].

## Conclusions

A focused genome analysis of almost 200 *B. pertussis* strains prevalent in the last 35 years in Japan revealed the existence of two major phylogenetic clades that coexist in the country. A shift in the population occurred in the past 10 years where isolates of the clade characterized by the *ptxA1 : ptxP3 : prn2* genotype began to dominate the *B. pertussis* population in Japan over isolates of the *ptxA2 : ptxP1 : prn1* genotype. This shift coincides with an increase in pertussis cases in the country.

Despite a lack of evidence for a direct effect of ACVs on the genotype shift of the *B. pertussis* population in Japan, this study noted a high rate of genetic variants for the genes targeted in the current ACV preparations, compared with other cell surface proteins. This observation confirms a certain degree of selective evolutionary pressure exerted by the ACVs.

Pertactin deficiency appeared around 1990 on at least two separate occasions, and probably again in the mid-1990s, in Japan, after ACVs had been adopted. Pertactin-deficient *prn1* isolates continue to be observed in Japan, although with decreasing prevalence since 2005.

Genetic variant analysis revealed a number of candidate proteins that are part of the *B. pertussis* core genome and may resist genetic variation, a subset of which consists of outer membrane proteins. In addition, several genes were identified that have a relatively high variant frequency, perhaps suggesting good antigenic properties. Such information may help in selecting candidate targets for a more effective pertussis vaccine.

## Data bibliography

1. Bart MJ, Harris SR, Advani A, Arakawa Y, Bottero D, *et al.* ENA SRA PRJEB2274 (2015).

## Supplementary Data

2370 Supplementary File 1
